# An Integrated Radiomics Model Incorporating Diffusion-Weighted Imaging and ^18^F-FDG PET Imaging Improves the Performance of Differentiating Glioblastoma From Solitary Brain Metastases

**DOI:** 10.3389/fonc.2021.732704

**Published:** 2021-08-30

**Authors:** Liqiang Zhang, Rui Yao, Jueni Gao, Duo Tan, Xinyi Yang, Ming Wen, Jie Wang, Xiangxian Xie, Ruikun Liao, Yao Tang, Shanxiong Chen, Yongmei Li

**Affiliations:** ^1^Department of Radiology, The First Affiliated Hospital of Chongqing Medical University, Chongqing, China; ^2^College of Computer & Information Science, Southwest University, Chongqing, China; ^3^Department of Nuclear Medicine, The First Affiliated Hospital of Chongqing Medical University, Chongqing, China; ^4^Department of Radiology, Chongqing United Medical Imaging Center, Chongqing, China; ^5^Department of Radiology, Chongqing General Hospital, Chongqing, China; ^6^Department of Oncology, People’s Hospital of Chongqing Hechuan, Chongqing, China

**Keywords:** ^18^F-FDG PET, diffusion-weighted imaging (DWI), apparent diffusion coefficient (ADC), glioblastoma, solitary brain metastases (SBM)

## Abstract

**Background:**

The effectiveness of conventional MRI (cMRI)-based radiomics in differentiating glioblastoma (GBM) from solitary brain metastases (SBM) is not satisfactory enough. Therefore, we aimed to develop an integrated radiomics model to improve the performance of differentiating GBM from SBM.

**Methods:**

One hundred patients with solitary brain tumors (50 with GBM, 50 with SBM) were retrospectively enrolled and randomly assigned to the training set (*n* = 80) or validation set (*n* = 20). A total of 4,424 radiomic features were obtained from contrast-enhanced T1-weighted imaging (CE-T1WI) with the contrast-enhancing and peri-enhancing edema region, T2-weighted imaging (T2WI), diffusion-weighted imaging (DWI)-derived apparent diffusion coefficient (ADC), and ^18^F-fluorodeoxyglucose positron emission tomography (^18^F-FDG PET) images. The partial least squares (PLS) regression with fivefold cross-validation is used to analyze the correlation between different radiomic features and different modalities. The cross-validity analysis was performed to judge whether a new principal component or a new feature dimension can significantly improve the final prediction effect. The principal components with effective interpretation in all radiomic features were projected to a low-dimensional space (2D in this study). The effective features of the new projection mapping were then sent to the random forest classifier to predict the results. The performance of differentiating GBM from SBM was compared between the integrated radiomics model and other radiomics models or nonradiomics methods using the area under the receiver operating characteristics curve (AUC).

**Results:**

Through the cross-validity analysis of partial least squares, hundreds of radiomic features were projected into a new two-dimensional space to complete the construction of radiomics model. Compared with the combined radiomics model using DWI + ^18^F-FDG PET (AUC = 0.93, *p* = 0.014), cMRI + DWI (AUC = 0.89, *p* = 0.011), cMRI + ^8^F-FDG PET (AUC = 0.91, *p* = 0.015), and single radiomics model using cMRI (AUC = 0.85, *p* = 0.018), DWI (AUC = 0.84, *p* = 0.017), and ^18^F-FDG PET (AUC = 0.85, *p* = 0.421), the integrated radiomics model (AUC = 0.98) showed more efficient diagnostic performance. The integrated radiomics model (AUC = 0.98) also showed significantly better performance than any single ADC, SUV, or TBR parameter (AUC = 0.57–0.71, *p* < 0.05). The integrated radiomics model showed better performance in the training (AUC = 0.98) and validation (AUC = 0.93) sets than any other models and methods, demonstrating robustness.

**Conclusions:**

We developed an integrated radiomics model incorporating DWI and ^18^F-FDG PET, which improved the performance of differentiating GBM from SBM greatly.

## Introduction

As the most common malignant brain tumor in adults, metastasis is estimated to be at least 10 times more common than primary malignant central nervous system tumors (1). Glioblastoma (GBM) accounts for more than half of all primary brain malignancies (2). Differentiating GBM from solitary brain metastases (SBM) preoperatively is significantly critical for optimizing individualized therapeutic decision-making, as the medical staging, therapeutic strategies, and prognosis are different (3–5). *En bloc* resection is preferred for metastases, and stereotactic radiosurgery is also considered an effective strategy for metastases of less than 3–4 cm (6), and maximal resection of the tumor followed by radiotherapy and temozolomide chemotherapy should be considered for GBM (7). Generally speaking, metastasis usually presents as multiple nodular enhancing lesions with surrounding edema in the cortical gray-white matter junction, whereas GBM mostly has general characteristic image features, such as the invasion of the deep white matter and the presence of solitary ring-enhancing lesion (3, 8). In patients with multiple lesions and systemic cancer, brain metastasis identification may be easily performed using conventional MRI (cMRI). However, when metastasis presents with a solitary ring-enhancing lesion or an unknown clinical history, it is challenging to differentiate the two tumors due to their similar imaging features. Both GBM and SBM can present with irregular ring enhancement and intratumoral necrosis on contrast-enhanced T1-weighted imaging (CE-T1WI), surrounding edema on T2-WI and ring-hypermetabolic on ^18^F-fluorodeoxyglucose positron emission tomography (^18^F-FDG PET) images. Histopathology is the gold standard for the diagnosis of GBM or metastasis. Unfortunately, the present way for identifying GBM from SBM is to undertake a biopsy or open surgical resection invasively. However, when the tumors are located near eloquent areas or the patient is weak, biopsy or open surgical resection may risk morbidity and mortality. Therefore, an accurate noninvasive preoperative method would be preferable and sometimes necessary (9, 10).

It has been shown that infiltrating neoplastic cells have been found in surrounding edema of GBM, while peritumoral edema of metastasis consists essentially of vasogenic edema, indicating that there are some differences in cells, edema type, angiogenesis, etc. between the peri-enhancing edema regions of the two tumors. However, the surrounding edema of the two tumors showed no enhancement, hypometabolic, or no obvious diffusion limitation. Therefore, it is reasonable to assume that there are pathophysiological abnormalities in the peritumoral edema area that cannot be visually recognized besides the tumor enhancement area. We hope to find a new and more effective method to distinguish two tumors based on the difference between the metastatic vasogenic edema and GBM infiltrative edema containing tumor cells infiltrating the white matter.

With the rapid development of medical image analysis, radiomics has become a hot research topic. Radiomics can noninvasively extract quantitative features of lesions from magnetic resonance images, providing important reference information for tumor characterizations, treatment monitoring, and outcome prediction (11). Previous studies have established radiomics models based on cMRI sequences to differentiate GBM from SBM and achieved good results. Qian et al. (12) developed a CE-T1WI-based radiomics model to differentiate GBM from SBM, with a test AUC value of 0.90. A radiomics model based on T1WI, T2WI, and CE-T1WI trained by Dong et al. (13) has a test AUC value of 0.76. Artzi et al. (14) established a radiomics model based on postcontrast 3D-T1W gradient echo images, and the test mean accuracy was 0.85. The radiomics classifier based on CE-T1WI established by Su et al. (15) yielded good performance with AUC values of 0.82 and 0.81 in the training and validation cohorts to distinguish GBM from SBM. These results are barely satisfactory for having limited value in demonstrating heterogeneity, function, and tumor metabolism and still have room for improvement. Some studies have reported that the mean diffusivity and minimum apparent diffusion coefficient (ADC) values of peritumoral edema seem lower in GBM than in SBM (16–18). Even though some other studies denied this result (19–21), diffusion-weighted imaging (DWI) seems to have the potential to distinguish GBM from SBM. Compared with cMRI, DWI can evaluate brain tumor diffusion and hypercellularity. ^18^F-FDG PET imaging has been shown to be helpful for assessing surgery and radiotherapy as well as providing important imaging biomarkers for tumor metabolism evaluation (22–26). Therefore, DWI and ^18^F-FDG PET imaging may show great potential for differentiating GBM from SBM. However, no studies have been reported to build a radiomics model incorporating DWI and ^18^F-FDG PET imaging in differentiating GBM from SBM.

We hypothesized that a multivariate radiomics model incorporating DWI and ^18^F-FDG PET could differentiate GBM from SBM more precisely than any other radiomics models or nonradiomics approaches, which will be more useful for clinicians to optimize clinical management decision-making. Thus, the study aimed to develop and validate a radiomics model using DWI and ^18^F-FDG PET to improve the performance of differentiating GBM from SBM.

## Methods

### Patient Enrollment

**Figure 1:**
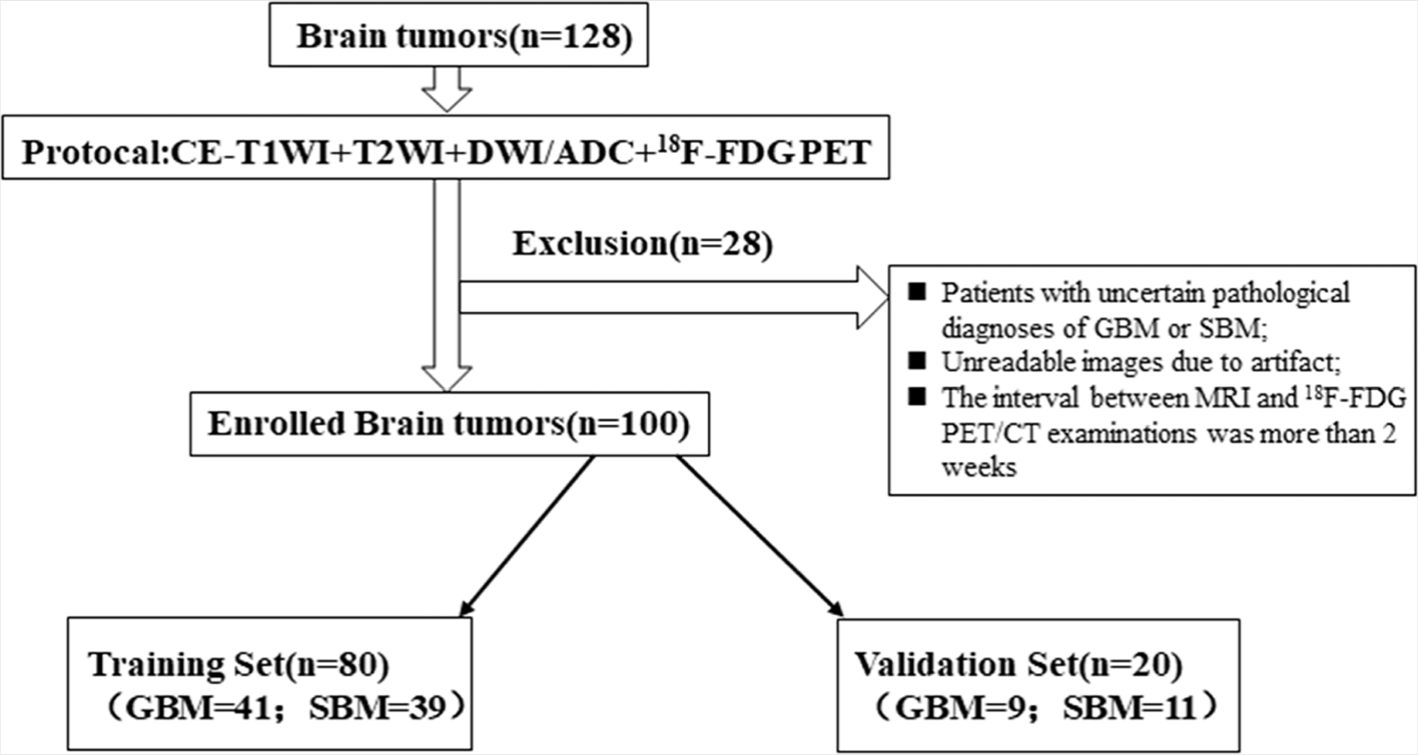
The flowchart of patient selection process.

This retrospective study was approved by the local institutional review board, and the informed consent was obtained. The data and pathological information were obtained from The First Affiliated Hospital of Chongqing Medical University and the United Medical Imaging Center. We identified 128 consecutive patients who were pathologically confirmed with GBM or SBM on surgical resection or biopsy performed at the Department of Neurosurgery of our hospital. The inclusion criteria were as follows: (1) pathologically confirmed GBM or SBM; (2) all the lesions are solitary and limited to a single lobe, not across the lobes; (3) performed CE-T1WI, T2WI, DWI, and ^18^F-FDG PET/CT examinations; (4) the interval between MRI and ^18^F-FDG PET/CT examinations was less than 2 weeks; and (5) no history of preoperative radiotherapy or other medical treatments before surgery. A total of 28 patients were excluded according to the exclusion criteria (**Figure 1**). Finally, a total of 100 consecutive patients were included in the study. The patient selection process is presented in a flowchart in **Figure 1** in detail.

### MR Imaging Acquisition

The MRI protocol for both training and validation sets included CE-T1WI, T2WI, DWI, and ^18^F-FDG PET imaging.

MR images were obtained from the 3.0-T MRI system (Genesis Signa and Signa HDtx) with an eight-channel head coil (GE Medical Systems, Chicago, IL, USA). The main parameters of the T2WI sequence were as follows: repetition time/echo time (TR/TE) = 8,000/140 ms, flip angle = 90°, slice thickness = 5 mm, acquisition matrix = 256 × 256. The main parameters of the CE-T1WI sequence were as follows: TR/TE = 750/15 ms, slice thickness = 5 mm, acquisition matrix = 384 × 256. The main parameters of the DWI sequence were as follows: TR/TE = 6,379/70 ms; section thickness = 5 mm; intersection gap = 1.5 mm; matrix size = 128 × 128; FOV = 260 × 260 mm. The apparent diffusion coefficient (ADC) map (*b* = 1,000) was generated from DWI images.

^18^F-FDG PET data acquisition was carried out with a PET/CT scanner (Philips Gemini TF 64 PET/CT scanner). The participants fasted for at least 6 h before ^18^F-FDG administration and stopped any drugs that could affect brain metabolism for at least 12 h before the ^18^F-FDG PET acquisition. Blood glucose levels were determined in all patients before ^18^F-FDG administration, and blood glucose level was less than 8.0 mmol/L. PET/CT images of the head were acquired 60 min after intravenous injection of 370–555 MBq ^18^F-FDG (produced by Sumitomo accelerator of Japan with a radiochemical purity of >95%). PET images were acquired for one bed position (5 min/bed position), and a slice thickness of 2 mm. Low-dose CT images were obtained with a standardized protocol of 400 mAs, 120 Kv, matrix size of 512 × 512, and a slice thickness of 1 mm. The fusion images (a slice thickness of 2 mm) were obtained by computer iterative reconstruction and attenuation correction.

### Image Preprocessing

For the CE-T1WI and T2WI data, signal intensity normalization was performed to reduce the variance in the T1-based signal intensity of the brain. We used the hybrid white-stripe method (22) for intensity normalization using the ANTsR and White Stripe packages (27, 28) in R, which incorporates processes of the statistical principles of image normalization, preserves ranks among the tissues, and matches the intensity of the tissues without upsetting the natural balance of the tissue intensities (29).

Skull stripping and tumor segmentation were performed by the 3D-Slicer Software (version 4.3, https://www.slicer.org) (30), an open-source software widely used for image visualization and segmentation. The tumor and perifocal edema contours were manually segmented using the fast-grow cut tool based on the T2WI imaging by two radiologists with 10 and 5 years of diagnostic experience, respectively, who were blinded to the final pathological result. The final region of interest (ROI) was determined by the two radiologists. If the divergence between segmentations was less than 5%, the final ROI was determined as the overlapping region of the two ROIs, otherwise, it was determined by the two radiologists. The segmented tumor contour was finally overlaid with source CE-T1WI, T2WI, ADC, and ^18^F-FDG PET image.

### Radiomics Feature Extraction

**Figure 2:**
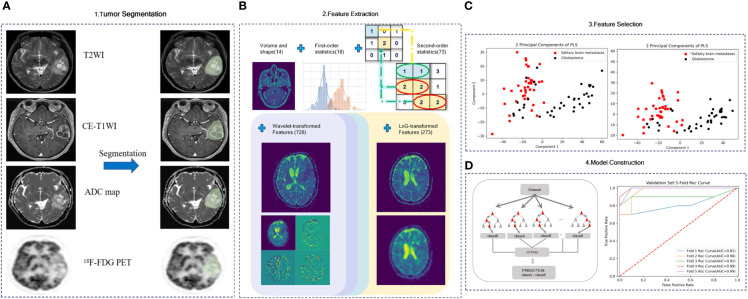
Radiomics worklist. **(A)** Part 1 includes image acquisition, registration, and segmentation. Signal intensity normalization is conducted for CE-T1WI and T2WI. **(B)** Part 2 includes the extraction of radiomics features. **(C)** Part 3 includes feature selection. **(D)** Part 4 includes model construction.

The radiomic features were composed of five groups of features: 18 first-order features, 14 shape features, 73 texture features, 273 LoG-transformed features, and 728 wavelet-transformed features. All patients had undergone CE-T1WI, T2WI, DWI, and ^18^F-FDG PET, from which 1,106 radiomic features were derived, respectively. Finally, all radiomic features were extracted for group comparisons after z transformed. The entire feature extraction algorithm was fully automated, which yielded identical features regardless of the operators. The overall process of the radiomics pipeline is shown in **Figure 2**.

### Feature Selection and Model Construction

In this study, we first used a *t*-test to screen radiomics features with significant independence, then applied partial least squares (PLS) to complete the regression analysis of those high-dimensional radiomics features in the training set. PLS regression method was applied using principal component analysis to extract and compress multiple independent variables X and multiple dependent variables Y into corresponding principal components U and V, respectively. Then, under the guidance of the canonical correlation principle and the multiple linear regression principle, the relationship can be analyzed between X and U, Y and V, and X and V. Thus, the relationship between X and Y can be studied. The PLS regression can project the high-dimensional data to the appropriate low-dimensional space and complete the effective feature selection of the data, which was chosen because the extracted radiomics features have the following two characteristics: (1) The number of feature dimensions extracted is far more than the number of patients. (2) Radiomics features exist in multiple correlations. PLS performs well in studies with small sample and multicollinearity among independent variables (radiomics features) and can emphasize the role of independent variables in the interpretation and prediction of dependent variables (classification of patients) when selecting feature vectors to remove the influence of useless noise on regression and make the radiomics model contain the least number of variables. As a result, final prediction performance gets better.

To evaluate the robustness of the radiomics model and consider the number of data samples, we applied fivefold cross-validation. Cross-validity analysis was used to determine the final output dimension size of PLS regression; after that, all radiomics features with significant independence were projected to the new two-dimensional space through PLS regression. Then a variety of classical classifiers were used to predict the outcome using the selected effective features and the optimal random forest classifier is selected to construct the final radiomics model. Finally, the predictive efficiency of the radiomics model was compared under the different modality features combination.

### Model Performance With Validation and Comparison of Diagnostic Performance

The accuracy of differentiating GBM from SBM using the above methods was assessed with the receiver operating characteristic (ROC) curve and the AUC values in two sets. The optimal thresholds of the AUC values were determined by maximizing the sum of the sensitivity and specificity values calculated for differentiating GBM from SBM.

The performance of the integrated radiomics model was compared with that of the three combined radiomics models, three single radiomics models, and five single nonradiomics methods. Bonferroni correction was applied to adjust the *p*-values for multiple comparisons. A Bonferroni-corrected significance level of *p* < 0.008 was used to compare the integrated radiomics model and six other radiomics models, and a value of *p* < 0.01 was used to compare between the integrated radiomics model and the five nonradiomics methods.

### Statistical Analysis

Statistical analyses were performed using R software (version 3.3.3). Differences in clinical information between the training and validation sets were evaluated using Student’s *t*-test and Chi-square tests, and *p* < 0.05 was considered statistically significant. The Student’s *t*-test was used to assess differences in the imaging parameters between GBM and SBM in the training and validation sets.

## Results

### Clinical Characteristics

All patients underwent biopsy or surgery, and their pathological examination results were assessed. Of the 80 enrolled patients in the training set, 41 (51.2%) were identified as GBM and 28 (36.8%) as SBM. Twenty patients in the validation set consisted of nine (45.0%) GBM and 11 (55.0%) SBM. The clinical characteristics of the training and validation sets are shown in **Table 1**. No significant differences were found between the patients with GBM and SBM regarding age and sex, which justified the applicability of the training and validation sets.

**Table 1:** Clinical characteristics of the patients.

Group	Training set			Validation set		
	GBM (*n* = 41)	SBM (*n* = 39)	*p*-Value	GBM (*n* = 9)	SBM (*n* = 11)	*p*-Value
Age	48.8 ± 11.2	49.6 ± 10.9	0.87	52.3 ± 10.3	51.8 ± 10.2	0.71
No of male patients	24 (58.5%)	17 (43.6%)	0.76	4 (44.4%)	5 (45.5%)	0.12
Biopsy	35 (85.4%)	31 (79.5%)		5 (55.6%)	7 (63.6%)	
Surgical resection	7 (14.6%)	8 (20.5%)		4 (44.4%)	4 (36.4%)	

Data are expressed as the mean ± standard deviation. Numbers in parentheses are percentages.

### Radiomics Feature Extraction

**Figure 3:**
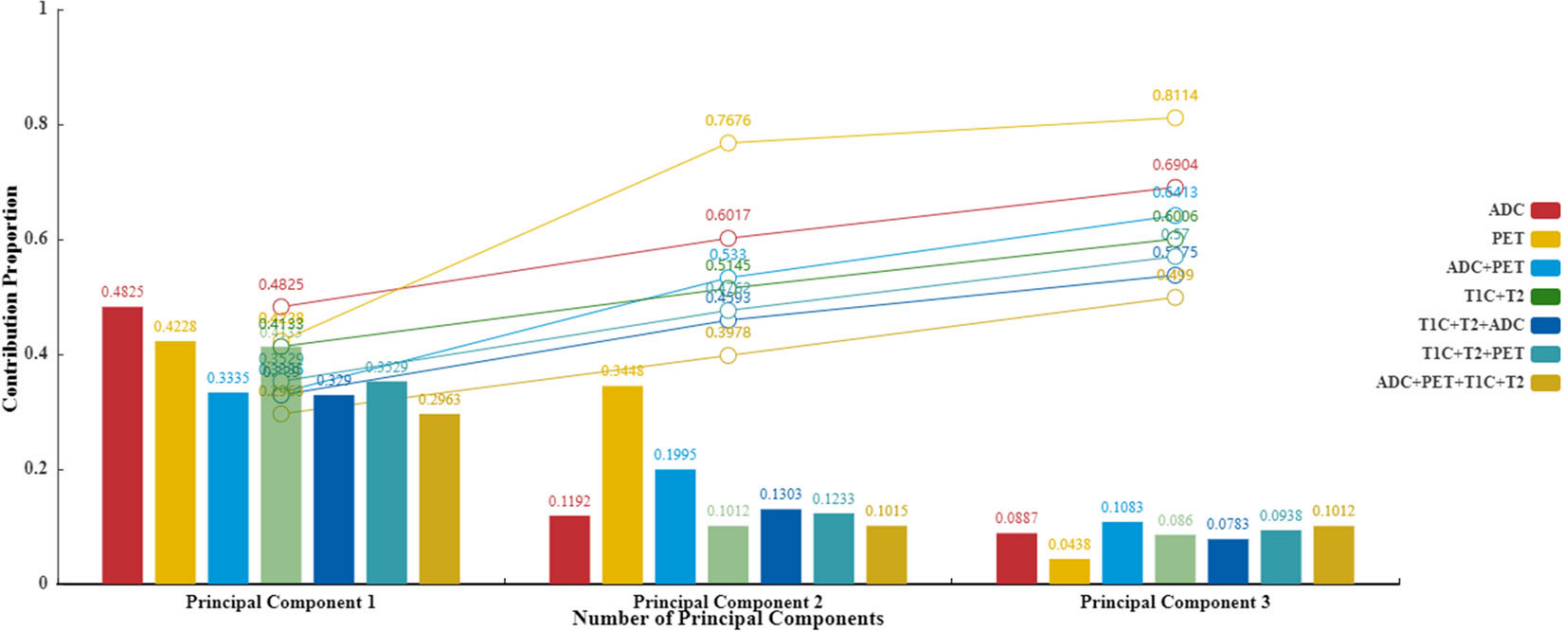
Principal component contribution histogram and cumulative contribution rate line chart.

In total, 4,424 radiomic features were extracted from the multiparametric MR data (1,106 features were derived from CE-T1WI, T2WI, ADC, and ^18^F-FDG PET). Partial least squares regression was used to find the correlation between radiomic features and patient classification. After cross-validity analysis, the top-m principal components of radiomic features with significant improvement for prediction results were selected by truncation method, so that hundreds of radiomic features were projected into a new m-dimensional space. **Table 2** shows the corresponding numerical relationship between different modality combinations (columns) and the final projection mapping dimensions (rows). The table starts from the analysis with only one principal component and gradually increases the number of retained principal components until the cross-validity principle is no longer satisfied, to select the number of final effective feature dimensions. The specific performance is the corresponding *Qh* ^2^ value less than 0.0975, indicating that adding a new principal component or feature dimension based on the previous number of principal components no longer has an obvious improvement on the final prediction effect and then ends the increase of the principal component number. It can be obtained from **Table 2** that the effective principal components of almost all modality combinations are less than or equal to 3. At the same time, judging from the importance and cumulative proportion of each principal component to the outcome (**Figure 3**), even when there are only three principal components, the newly screened features can affect the final result by more than 50%. From this point of view, it is reasonable to use the partial least squares method to screen the effective features. Through the experimental test, the number of dimensions of the optimal result is 2.

**Table 2:** Score of crossvalidity analysis (*Qh* ^2^ score).

Modality combination	Number of principal components					Effective number
	1 Component	2 Components	3 Components	4 Components	5 Components	
ADC	1	0.2402	0.0498	None	None	2
PET	1	−0.0122	None	None	None	1
ADC+PET	1	0.1755	0.1990	−0.0559	None	3
T1+T2	1	0.1115	0.1243	0.1260	0.0369	4
T1+T2+ADC	1	0.0839	None	None	None	1
T1+T2+PET	1	0.2057	0.0183	None	None	2
T1+T2+ADC+PET	1	0.1829	0.0475	None	None	2

Qh^2^≤0.0975indicates that adding a new principal component or feature dimension based on the previous number of principal components no longer has an obvious improvement effect on the final prediction effect, and then ends the increase of the component number.

**Figure 4:**
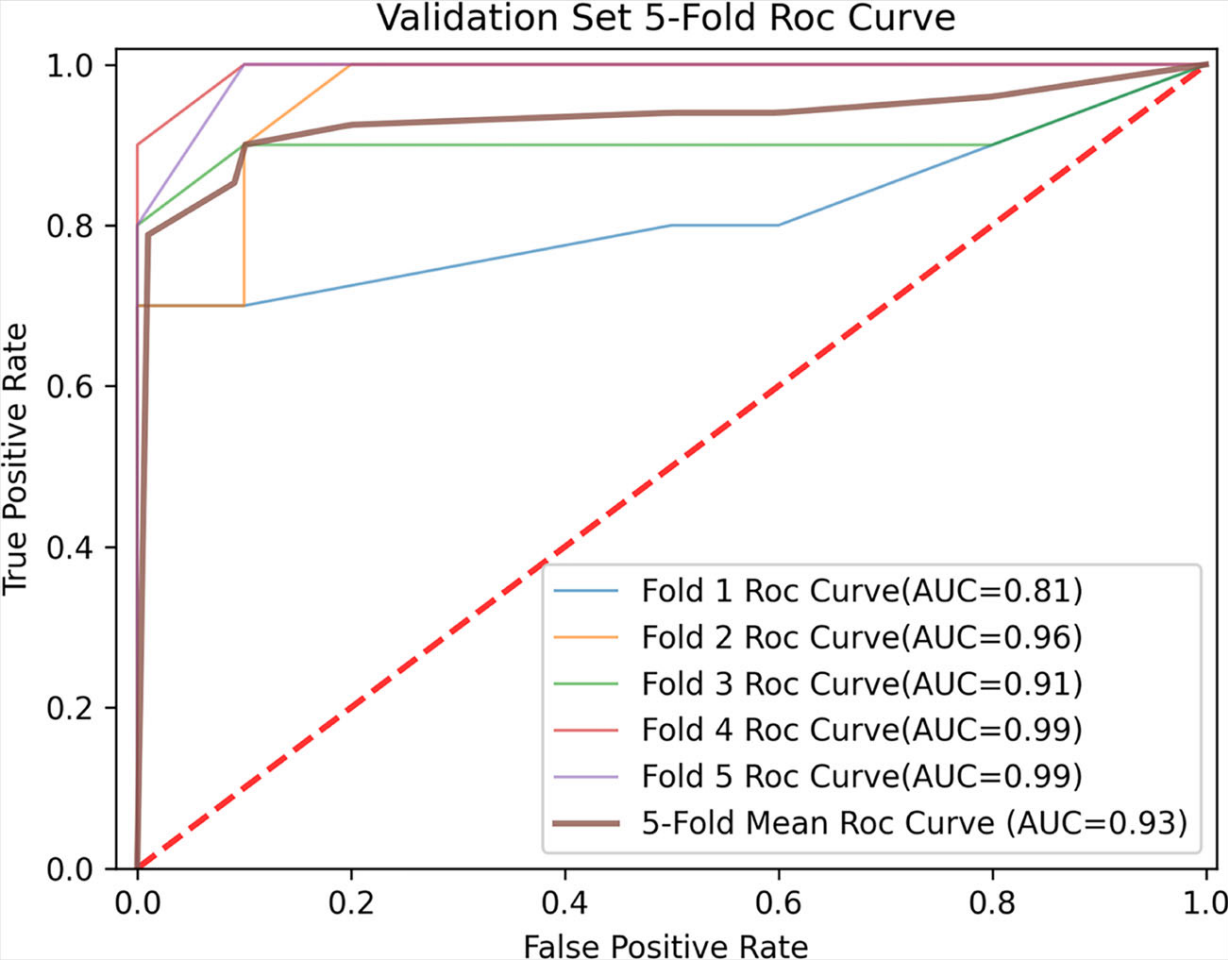
Fivefold and mean receiver operating characteristic (ROC) curve for prediction in the validation cohort.

**Figure 5:**
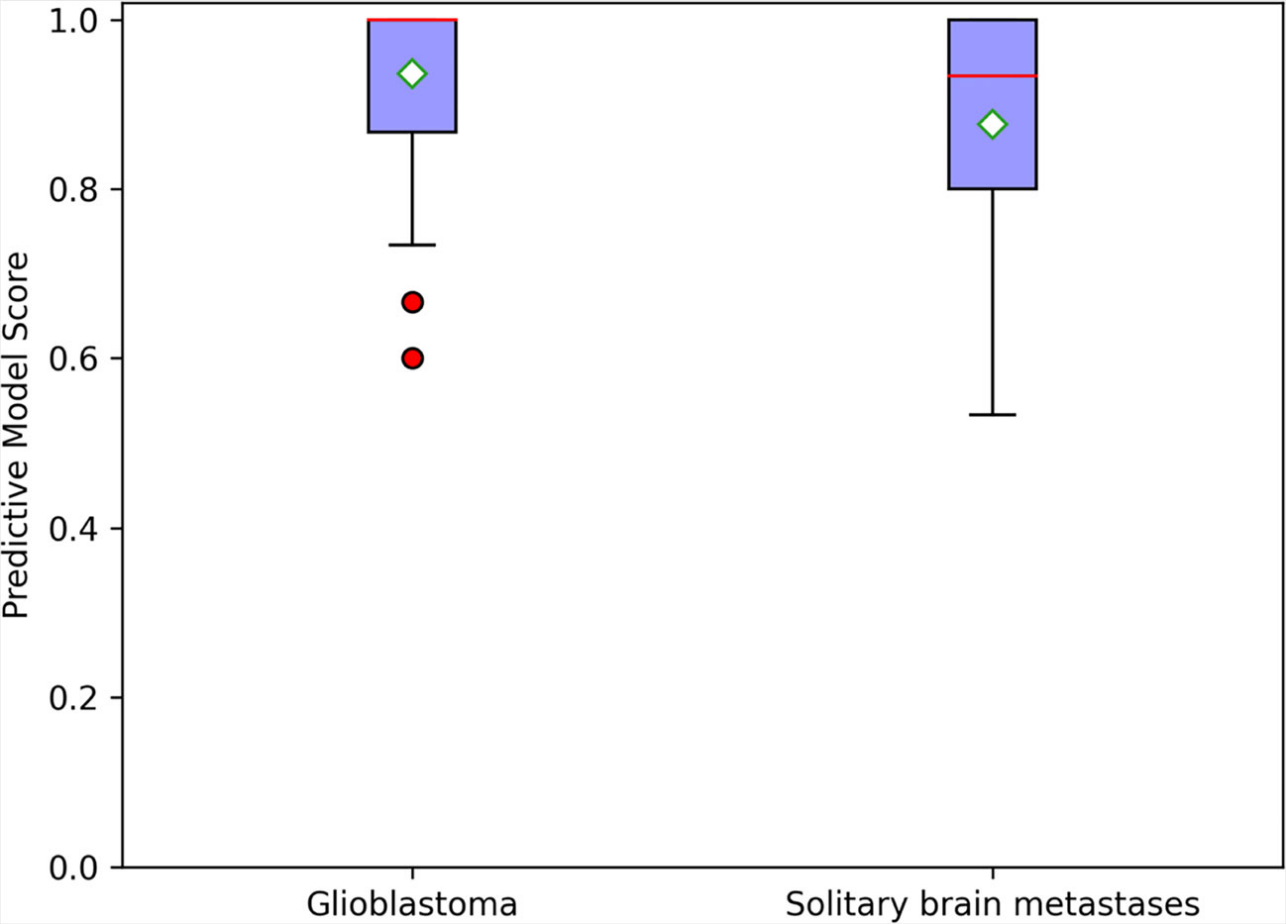
Random forest classifier scores for glioblastoma and solitary brain metastases in the validation cohort; the red line indicates median, and the white diamond represents average prediction score.

In constructing a random forest model, not all the training data were used by each decision subtree, so these data can be used as test cases to measure the generalization performance of the model by calculating the classification error of out-of-bag estimation, which was 0.10 in the training cohort. The mean prediction accuracy of 84.00% (AUC = 0.9330) for fivefold cross-validation was achieved in the validation cohort (**Figure 4**). For a random forest classifier, the classification score matrix represents the possibility that the label comes from a specific category. As shown in **Figure 5**, the red line indicates the median, and in our radiomics classifier model, the average scores (white diamond in **Figure 5**) of correct prediction for samples with GBM label and SBM label are 0.9365 (95% CI: 0.9044–0.9686) and 0.8762 (95% CI: 0.8350–0.9174), respectively.

### Model Performance and Comparison of Diagnostic Performance

The sensitivity and specificity of the integrated radiomics model in the training set were 92.5% and 98.7%, respectively. The AUC value was higher in the integrated radiomics model (AUC = 0.98) than in the combined radiomics models (DWI + ^18^F-FDG PET: AUC = 0.93, *p*=0.014; Conventional + DWI: AUC = 0.89, *p* = 0.011; Conventional + ^8^F-FDG PET: AUC = 0.91, *p* = 0.015), the single radiomics model using cMRI (AUC = 0.85, *p* = 0.018), DWI (AUC = 0.84, *P* = 0.017), ^18^F-FDG PET (AUC = 0.85, *p* = 0.421) and single nonradiomics method (AUC = 0.57–0.71, *p* < 0.05), showing the integrated model with better performance (**Table 3**).

**Table 3:** Comparison of diagnostic performance between integrated radiomics model and other methods in the training and validation sets.

Group		Training set				Validation set				
		AUC	*p*-Value*	Sensitivity	Specificity	AUC	Sensitivity	Specificity	
Integrated radiomics model	Conventional + DWI+ ^18^F-FDG PET	0.98 (0.93, 0.99)		92.5%	98.7%	0.93 (0.81, 0.97)	83.5%	84.9%	
Combined radiomics model	DWI + ^18^F-FDG PET	0.93 (0.89,0.97)	0.014	82.3%	91.2%	0.81 (0.67,0.89)	72.5%	78.1%	
	Conventional + DWI	0.89 (0.83, 0.94)	0.011	92.1%	89.7%	0.86 (0.74, 0.93)	76.1%	86.8%	
	Conventional + ^18^F-FDG PET	0.91 (0.84, 0.95)	0.015	91.7%	94.7%	0.83 (0.74, 0.93)	80.4%	80.3%	
Single radiomics model	Conventional MR	0.85 (0.74, 0.93)	0.018	82.6%	88.7%	0.84 (0.77, 0.91)	79.8%	76.1%	
	DWI	0.84 (0.71, 0.87)	0.017	77.2%	75.8%	0.83 (0.78, 0.88)	82.2%	74.5%	
	^18^F-FDG PET	0.85 (0.72, 0.91)	0.421	66.4%	93.5%	0.84 (0.76, 0.89)	87.8%	72.2%		
Single nonradiomics method	ADC max	0.59 (0.52, 0.65)	<0.001	56.4%	62.1%	0.51 (0.49, 0.72)	77.1%	61.3%	
	ADC avg	0.57 (0.51, 0.63)	<0.001	61.4%	72.3%	0.53 (0.51, 0.64)	67.3%	77.3%	
	SUV max	0.67 (0.62, 0.75)	<0.001	64.1%	53.4%	0.55 (0.47, 0.62)	62.1%	57.1%	
	SUV avg	0.64 (0.59, 0.71)	<0.001	81.7%	74.3%	0.59 (0.56, 0.67)	69.3%	62.7%	
	TBR max	0.71 (0.66, 0.77)	<0.001	80.4%	71.9%	0.67 (0.63, 0.77)	71.2%	89.3%	

Numbers in parentheses are 95% confidence intervals.

ADC, apparent diffusion coefficient; SUV, standardized uptake value; TBR, tumor-to-background ratio.

*P-value refers to the significance among the differences of the AUCs between the integrated radiomics model and the other model or method.

**Figure 6:**
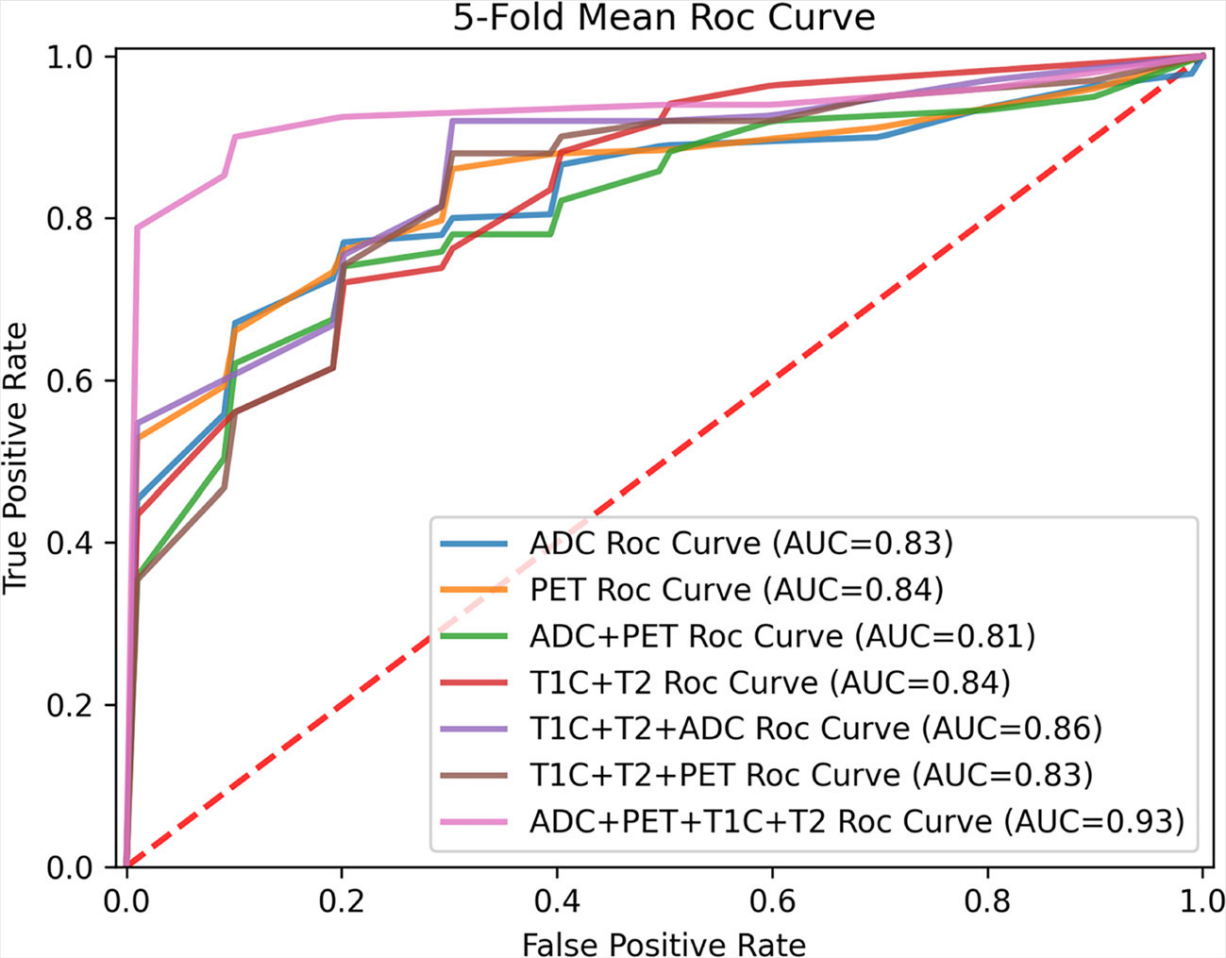
Fivefold mean ROC curve for different modality combination.

The sensitivity and specificity of the integrated radiomics model in the validation set were 83.5% and 84.9%, respectively. The AUC value was higher in the integrated radiomics model (AUC = 0.93) than in the combined radiomics models (DWI + ^18^F-FDG PET: AUC = 0.81; Conventional + DWI: AUC = 0.86; Conventional + ^8^F-FDG PET: AUC = 0.83), the single radiomics model using cMRI (AUC = 0.84), DWI (AUC = 0.83), and ^18^F-FDG PET (AUC = 0.84) and the single nonradiomics method (AUC = 0.51–0.67), showing the integrated model with better performance as well. The comparison of diagnostic performance of the radiomics models in the validation is shown by the fivefold mean ROC curve for different combinations in **Figure 6**, and more evaluation indicator information can be seen in **Table 4**.

**Table 4:** Comparison of more evaluation indicator information.

Modality combination	Evaluating indicator				
	ACC	Sensitivity	Specificity	PPV	NPV
ADC	0.76	0.828	0.745	0.7775	0.7565
PET	0.7699	0.878	0.722	0.7823	0.7695
ADC+PET	0.75	0.725	0.781	0.7710	0.7560
T1+T2	0.74	0.798	0.761	0.7388	0.7561
T1+T2+ADC	0.8099	0.761	0.868	0.8603	0.8085
T1+T2+PET	0.80	0.804	0.803	0.8191	0.8323
T1+T2+ADC+PET	0.84	0.835	0.849	0.8701	0.8728

## Discussion

In the present study, we built seven radiomics models and five nonradiomics methods and compared their performance. By optimizing the radiomics models from single parameter, single and double sequences to multimodality, we finally concluded that the integrated radiomics model incorporating DWI and ^18^F-FDG PET outperformed any other radiomics models and nonradiomics methods. The integrated radiomics models in the training and validation sets have an AUC of 98% and 93%, a sensitivity of 92.5% and 83.5%, and a specificity of 98.7% and 84.9%, respectively. Some studies have used advanced imaging modalities (DWI and PET, etc.) to differentiate GBM from SBM. Lee et al. (29) analyzed patient age and sex, minimum ADC value, and ADC ratio of the two groups and found a statistical difference between GBM and metastasis. Kamson et al. (31) tested the accuracy of α[^11^C]-methyl-l-tryptophan (AMT)–positron emission tomography (PET) to differentiate GBM from metastases and concluded that tumor/cortex AMT SUV ratios could distinguish GBM from metastases. However, their researches are just quantitative or semi-quantitative statistical analyses and limited to depict heterogeneous nature and excavate deeper information of GBM and metastases. As far as we know, this is the first radiomic study in brain tumors that combines MRI and ^18^F-FDG PET. The CE-T1WI/T2WI, DWI, and ^18^F-FDG PET in the model provided structural, functional, and metabolic information at the same time and space, which makes our research more comprehensive and in-depth.

This study extended previous radiomic studies that only extracted features from cMRI sequences on enhancing tumor region or peri-enhancing edema region to differentiate GBM from SBM (12–15, 32–35). First, we incorporated DWI and ^18^F-FDG PET based on cMRI sequences, which is the first of its kind. Second, the ROI of our study covers both enhancing tumor region and peri-enhancing edema region, which is larger than that of previous studies and is conducive to extracting more effective features. Third, we used the *t*-test to screen radiomics features with significant independence, and then used partial least squares regression to process these features further. The partial least squares regression with fivefold cross-validation was applied to analyze the correlation between different radiomics features within and between different imaging types in this study. Through cross-validity analysis to determine the final output dimension size of PLS regression, all radiomics features were mapped to the new two-dimensional space. After that, a variety of classical classifiers were used to predict classification outcomes using the new effective features, and the classifier with the best result (The random forest classifier is selected in this paper) will be selected to build the final radiomics model. Unlike the partial original radiomics features screened by LASSO in previous studies, we built the new interpretation dimensions according to the correlation between all radiomics features principal components and the corresponding label principal components to complete the construction of radiomics model. Even in a few new projection mapping dimensions, the final results are satisfactory. By comparing the diagnostic performance of models, we finally found an optimal integrated radiomics model to distinguish GBM from SBM. The integrated radiomics model achieved a noteworthy result, with AUCs of 0.98 (95% CI: 0.83–0.99) and 0.93 (95% CI: 0.81–0.97) in the training and validation sets, respectively, indicating the higher predictive performance of our study than the former ones.

Nevertheless, there are several limitations in this study. The total sample size was relatively small for the radiomics study, and a larger data set is needed to assess and adjust our model. Moreover, the validation set size is small, leading to the relatively low sensitivity of the integrated radiomics model. Finally, the study is a retrospective single-center study, and larger data sets from multicenter registration using different MR protocols should be interrogated to improve the radiomics model’s stability further. If validated correctly and properly, this integrated model is expected to differentiate GBM from SBM before surgery, which can improve the diagnostic accuracy and provide help for the treatment plan and prognostic evaluation. Although the low-dimensional effective features screened by PLS can get a satisfactory performance, it ignores those principal components with large numbers but small contributions in feature screening, which may lead to a low cumulative contribution rate of the final screened effective features. Therefore, how to mine new information from these principal components with a large number but small contribution can be the future research direction.

In conclusion, our results confirm that the integrated radiomics model incorporating functional (DWI) and metabolic (^18^F-FDG PET) sequences can achieve promising diagnostic efficiency for distinguishing between GBM and SBM with robustness. A large-scale multicenter study should be carried out to further confirm the preliminary results, thus making this noninvasive, simple and effective method applicable for routine clinical practice.

## Data Availability Statement

The raw data supporting the conclusions of this article will be made available by the authors, without undue reservation.

## Ethics Statement

The studies involving human participants were reviewed and approved by The First Affiliated Hospital of Chongqing Medical University. The patients/participants provided their written informed consent to participate in this study. Written informed consent was obtained from the individual(s) for the publication of any potentially identifiable images or data included in this article.

## Author Contributions

Manuscript writing and statistical analysis: LZ, RY, and JG. Manuscript editing, supervised image segmentation, and statistical analysis: RY. Image postprocessing, data provision, and informatics software support: DT and LZ. Supervised image postprocessing: JG, DT, and XY. Pathologic analysis: LZ, MW, YL, and SC. Clinical/oncologic database curation and oversight: YL, SC, and LZ. Database construction and data provision: XX, RL, SC, and JW. Database construction, clinical oversight, conceptual feedback, and project integrity: JG, DT, TY, YL, and SC. All authors contributed to the article and approved the submitted version.

## Funding

This work was supported by the National Natural Science Foundation of China (31800823), the Medicine Scientific key Research Project of Chongqing Municipal Health and Family Planning Commission (2016ZDXM002), the Chongqing basic research and frontier exploration project of Chongqing Science & Technology Commission (cstc2018jcyjAX0584), the National Key Research & Development Plan of Ministry of Science and Technology of the People’s Republic of China (2016YFC0107109). We thank the funders supporting the research.

## Conflict of Interest

The authors declare that the research was conducted in the absence of any commercial or financial relationships that could be construed as a potential conflict of interest.

## Publisher’s Note

All claims expressed in this article are solely those of the authors and do not necessarily represent those of their affiliated organizations, or those of the publisher, the editors and the reviewers. Any product that may be evaluated in this article, or claim that may be made by its manufacturer, is not guaranteed or endorsed by the publisher.
